# Supplementary material for: Patterns of Intron Gain and Loss in Fungi
Source: PLoS Biol. 2004 Nov 30;2(12):e422. doi: 10.1371/journal.pbio.0020422 (PMC532390; doi:10.1371/journal.pbio.0020422)
Supplement: Table S1 — Also available at http://genes.mit.edu/NielsenEtAl/. (4.3 MB ZIP). [file pbio.0020422.st001.zip › NielsenEtAl/html/308.html]

AN4281.1.NCU08477.1.MG06962.1.FG10873.1


```
 CLUSTAL W (1.82) Multiple Sequence Alignments - Introns Inserted


Sequence 1: MG06962.1	202 aa
Sequence 2: FG10873.1	202 aa
Sequence 3: AN4281.1	200 aa
Sequence 4: NCU08477.1	243 aa
Alignment Length: 243 aa
Number Identitical Residues: 176 aa
Alignment Score (without introns) 6588


MG06962.1 	----------------------------------------MNPE2YDYLFKLLLIGDSGV
NCU08477.1	MLTCLTFRVPTASPLHPTSSPSLSRLDRSPPPPPPKNPADDATR~YDYLFKLLLIGDSGV
FG10873.1 	----------------------------------------MNPE2YDYLFKLLLIGDSGV
AN4281.1  	-----------------------------------------MVR~YDYLFKLLLIGDSGV
          	                                           . ***************

MG06962.1 	GKSCLLLRFADDTYTESYISTIGVDF0KIRTIELDGKTVKLQI0WDTAGQERFRTITSSY
NCU08477.1	GKSCLLLRFADDTYTESYISTIGVDF0KIRTIELDGKTVKLQI0WDTAGQERFRTITSSY
FG10873.1 	GKSCLLLRFADDTYTESYISTIGVDF0KIRTIELDGKTVKLQI0WDTAGQERFRTITSSY
AN4281.1  	GKSCLLLRFADDTYTESYISTIGVDF0KIRTIELDGKTVKLQI0WDTAGQERFRTITSSY
          	************************** **************** ****************

MG06962.1 	YRGAHGICVVYDVTDMDSFNNVKQWLQEIDRYATEGVNKLLVGNKSDMADKKVVDYTVAK
NCU08477.1	YRGAHGICVVYDVTDMDSFNNVKQWLQEIDRYATEGVNKLLVGNKSDMTDKKVVEYTVAK
FG10873.1 	YRGAHGICVVYDVTDMDSFNNVKQWLQEIDRYATEGVNKLLVGNKSDMSDKKVVEYSVAK
AN4281.1  	YRGAHGICVVYDVTDMDSFNNVKQWLQEIDRYATEGVNKLLVGNKSDMEDKKVVEYTVAK
          	************************************************ *****:*:***

MG06962.1 	0EFADSLGIPFLETSAKNANNVEQAFLTMARQIKERMG-TTTTNNSKPSVQVGPGQGVGS
NCU08477.1	0EFADSLGIPFLETSAKNASNVEQAFLTMARQIKERMGSSIATNNTKASVNVSPGHGVSN
FG10873.1 	0EFADSLGIPFLETSAKNASNVEQAFLTMARQIKERMG-TTTANNTKPSVQVGQGQGVGS
AN4281.1  	0EFADSLGIPFLETSAKNASNVEQAFLTMARQIKERMG-TATVNN-KPTVQVGQGQGVQS
          	 ******************.****************** : :.** *.:*:*. *:** .

MG06962.1 	NQSNSCC
NCU08477.1	NSSGGCC
FG10873.1 	SSNNSCC
AN4281.1  	GSAGGCC
          	.. ..**
```
